# Less Is More: Optimized pharmacotherapy with improved coNtinuity of CarE in hospitaLized oLder peOple (LIMONCELLO): study protocol of a cluster randomized controlled trial

**DOI:** 10.1186/s12877-025-06533-0

**Published:** 2025-12-24

**Authors:** Sjacky Cooijmans, Eline M. C. Engelen, Stéphanie C. M. Wuyts, Cornelis Kramers, Michiel A. van Agtmael, Patricia M. L. A. van den Bemt, Marcel L. Bouvy, Hayat Amouch, Reshma S. Autar, Guillemette E. Benoist, Inge R. F. van Berlo-van de Laar, Judith Y. M. N. Derijks-Engwegen, Geke van den Elsen, Frouke M. Engelaer, Kim B. Gombert-Handoko, David R. M. Jansen, Marieke H. M. Kerskes, Willemien J. Kruik-Kollöffel, Marianne A. Kuijvenhoven, Hugo M. van der Kuy, Geert Labots, Eveline P. van Poelgeest, Roos Sablerolles, Bastiaan T. G. Sallevelt, Feikje van Stiphout, Bert N. Storm, Margot Taks, Patty J. I. Teeuwisse, Jonne Uiterwijk, Ariël Vondeling, Merijn de Vries, Elsbeth Wesselink, Nikki M. L. Wiggers, Hugo A. J. M. de Wit, Henk J. Schers, Eddy M. M. Adang, Madelon van Wely, Noortje van Herwaarden, Wilma Knol

**Affiliations:** 1 Department of Pharmacy, Pharmacology and Toxicology, Radboud University Medical Center, Mailbox 9101, Geert Grooteplein-Zuid 10, (Route Number 456), 6500 HB Nijmegen, The Netherlands; 2 Department of Internal Medicine, Amsterdam University Medical Center – location VUmc, Amsterdam, The Netherlands; 3 Research and Expertise Centre in Pharmacotherapy Education (RECIPE), Amsterdam, The Netherlands; 4 Research Centre for Digital Medicine, Faculty of Medicine and Pharmacy, Vrije Universiteit Brussel, Brussels, Belgium; 5 Department of Internal Medicine, Radboud University Medical Center, Nijmegen, The Netherlands; 6 Department of Clinical Pharmacy, Canisius Wilhelmina Hospital, Nijmegen, The Netherlands; 7 Department of Clinical Pharmacy and Pharmacology, University Medical Center Groningen, Groningen, The Netherlands; 8 Division of Pharmacoepidemiology and Clinical Pharmacology, Utrecht Institute for Pharmaceutical Sciences, Utrecht University, Utrecht, The Netherlands; 9 Department of Clinical Pharmacy, Amphia Ziekenhuis, Breda, The Netherlands; 10 Department of Pharmacy, Deventer Ziekenhuis, Deventer, The Netherlands; 11 Department of Clinical Geriatrics, Ziekenhuisgroep Twente, Almelo, The Netherlands; 12 Department of Internal Medicine, Leiden University Medical Center, Leiden, The Netherlands; 13 Department of Clinical Pharmacy and Toxicology, Leiden University Medical Center, Leiden, The Netherlands; 14 Department of Geriatrics, Radboud University Medical Center, Nijmegen, The Netherlands; 15 Department of Clinical Pharmacy, Catharina Ziekenhuis, Eindhoven, The Netherlands; 16 Department of Clinical Pharmacy, Ziekenhuisgroep Twente, Almelo and Hengelo, The Netherlands; 17 Pharmacy & Clinical Pharmacology, Amsterdam University Medical Center, Amsterdam, The Netherlands; 18 Department of Hospital Pharmacy, Erasmus MC University Medical Center, Rotterdam, The Netherlands; 19 Department of Internal Medicine, HagaZiekenhuis, Den Haag, The Netherlands; 20 Department of Internal Medicine, Geriatrics, Amsterdam University Medical Centers, Amsterdam, The Netherlands; 21 Clinical Pharmacy, University Medical Center Utrecht, Utrecht University, Utrecht, The Netherlands; 22 Department of Geriatric Medicine, Meander Medical Center, Amersfoort, The Netherlands; 23 Department of Hospital Pharmacy, HagaZiekenhuis, Den Haag, The Netherlands; 24 Department of Pharmacy and Clinical Pharmacology, Amsterdam University Medical Center Location AMC, Amsterdam, The Netherlands; 25 Department of Clinical Geriatrics, Diakonessenhuis, Utrecht, Zeist and Doorn, The Netherlands; 26 Department of Clinical Pharmacy, Meander Medical Center, Amersfoort, The Netherlands; 27 Department of Clinical Pharmacy, Zaans Medical Center, Zaandam, The Netherlands; 28 Department of Primary and Community Care, Radboud University Medical Center, Nijmegen, The Netherlands; 29 Department for Health Evidence, Radboud University Medical Center, Nijmegen, The Netherlands; 30 Center for Reproductive Medicine, Amsterdam Reproduction & Development Research Institute, Amsterdam University Medical Center, University of Amsterdam, Amsterdam, The Netherlands; 31 Department of Rheumatology, Sint Maartenskliniek, Nijmegen, The Netherlands; 32 Department of Geriatric Medicine and Expertise Centre Pharmacotherapy in Older People, University Medical Center Utrecht, Utrecht University, Utrecht, The Netherlands

**Keywords:** Polypharmacy, Drug-related readmissions, Medication review, Older persons, Inappropriate prescribing, Cluster randomized controlled trial

## Abstract

**Background:**

Polypharmacy is associated with negative health outcomes including drug-related hospital admissions, particularly in older patients. Approximately half of drug-related (re)admissions are potentially preventable, and are therefore a target for healthcare interventions. Several studies have investigated the effects of in-hospital medication reviews on clinically relevant outcome measures, such as drug-related readmissions (DRAs). The identified factors for success of the intervention were multicomponent interventions, multidisciplinary approaches, attention to transitional care and the selection of high-risk patients. In the present study, the impact of transitional multidisciplinary pharmacotherapeutic care (TMPC) on DRAs in a high-risk population is assessed.

**Methods:**

Less Is More: Optimized pharmacotherapy with improved coNtinuity of CarE in hospitaLized oLder peOple (LIMONCELLO) is a cluster randomized controlled trial. A cluster is defined at the hospital level, and each cluster is randomly allocated (1:1) to the intervention or control group stratified by university or general hospitals. Patients aged 70 years and older with polypharmacy, a current non-elective hospital admission, completed medication reconciliation, and an increased risk of a DRA, as determined by the use of a DRA prediction model, are selected. TMPC consists of four elements: a pharmacotherapeutic analysis, a transitional multidisciplinary discussion, a pharmacotherapeutic care interview including shared decision-making with the patient, and a discharge letter with the pharmacotherapeutic plan sent to the general practitioner and community pharmacist. The comparator is usual care provided in the participating control hospitals. The primary outcome is the proportion of patients with DRA in the first 30 days after discharge. The secondary outcomes include cost-effectiveness, quality of life, and mortality.

**Discussion:**

The novelty of this study lies in the selection of a patient group expected to be at high risk for DRAs, on the basis of an evidence-based prediction model, as well as in the combination of components used in the intervention. By selecting high-risk patients and applying a multicomponent approach, this study is expected to increase current knowledge on the effects of TMPC on clinically relevant outcomes and cost-effectiveness in hospitalized older patients.

**Trial registration:**

Clinicaltrials.gov: NCT05899114, prospective submission, registration June 02 2023 (first inclusion June 5 2023).

**Supplementary Information:**

The online version contains supplementary material available at 10.1186/s12877-025-06533-0.

## Background

In the rapidly growing aging population, multimorbidity increasingly leads to polypharmacy, usually defined as the concomitant and chronic use of five or more medications [1]. Almost half of the people aged 65 years or older in high-income countries have polypharmacy [2, 3]. Polypharmacy can lead to undesired health outcomes, such as an increased risk of emergency department (ED) visits and unplanned hospital admissions [4–6]. Approximately 10–30% of all acute hospital admissions of patients aged 65 years and older are drug-related [7–9]. A significant portion of these admissions are readmissions [5, 9]. A drug-related readmission (DRA) is a second hospital admission that occurs after a previous hospitalization, in which pharmacotherapy or a lack of appropriate pharmacotherapy according to guidelines is the main cause [9, 10]. Nearly half of the drug-related (re)admissions are potentially preventable [9, 11], representing an important modifiable factor contributing to morbidity and mortality in older adults. Unfortunately, despite multiple efforts to reduce the incidence of (preventable) drug-related admissions in the Netherlands [12], the numbers have not decreased [7, 13]. To reduce the occurrence of drug-related (re)admissions in older adults and, consequently, healthcare costs and to improve the quality of care, the development and implementation of effective medication optimization strategies are needed. One of these strategies is performing a medication review in older adults. A medication review is defined as a *“structured evaluation of a patient’s medicines with the aim of optimizing medicines use and improving health outcomes. This entails detecting drug-related problems (DRPs) and recommending interventions.”* [14]. Medication reviews can vary in type and are executed in various care settings.

The effects of medication review interventions on clinically relevant outcome measures are the subject of debate, and the evidence is inconsistent. While some studies demonstrate beneficial effects on severe negative outcomes such as hospital readmissions, falls, and mortality, other studies do not find these effects [15–18]. A scoping review of systematic reviews containing different types of studies by Craske et al. revealed that the evidence concerning the effectiveness of medication reviews was uncertain across different care settings and patient populations [15, 18]. A recent Cochrane review of randomized trials concluded that medication reviews in hospitalized older adults likely reduce the risk of hospital admissions and may reduce ED contacts, but the most effective type of medication review remains inconclusive [15].

The lack of clarity regarding the effectiveness of medication review interventions in previous studies is partially explained by heterogeneous study designs, potentially caused by the absence of a gold standard for conducting a medication review [17]. The scopes of these reviews range from basic medication list revisions to comprehensive medication review interventions incorporating multiple components [15]. Previous studies have demonstrated that intensifying the intervention by adopting a multicomponent approach may be more effective than implementing medication reviews as isolated interventions [19–21]. The suggested effective components include medication reconciliation, patient education, and improved transitional care [20]. Transitional care involves care during the movement of a patient from one healthcare setting to another. To ensure smooth transition, multidisciplinary collaboration is essential [21]. Therefore, medication review interventions should incorporate these described components, with a specific focus on the transition of care. Another contributing factor to the lack of clarity on effectiveness is the variation in the studied population. Frequently, general populations of older people with polypharmacy are selected as study population. However, previous studies have shown that selecting high-risk populations results in a lower number needed to treat to prevent hospital readmissions, as these persons are at elevated risk of unfavourable outcomes such as readmissions, and ED contacts which might increase the likelihood of detecting an effect on the selected outcome [15, 22]. Furthermore, the effects of medication reviews have been studied across different care settings, including both primary and secondary care. Performing medication review interventions during hospitalization seems particularly effective because of the higher baseline risk of DRA after hospitalization [23]. This is partly due to the hospital’s high-risk setting which is prone to medication errors, which can result from changes in the medication regimen by multiple prescribers, changes in patients’ medical conditions, and lack of communication during care transitions between hospital and outpatient healthcare professionals [15, 24].

The Less Is More: Optimized pharmacotherapy with improved coNtinuity of CarE in hospitaLized oLder peOple (LIMONCELLO)-trial will take all the factors mentioned above into account. This study pays special attention to the ‘high-risk subgroup’ within the population of older adults with polypharmacy, and focuses on improved transitional care. The purpose of this study is to investigate the effects of a multicomponent medication review intervention called transitional multidisciplinary pharmacotherapeutic care (TMPC) on DRAs, compared with usual care, in hospitalized older patients with a high risk of DRAs.

## Methods

### Study design and setting

**Fig. 1:**
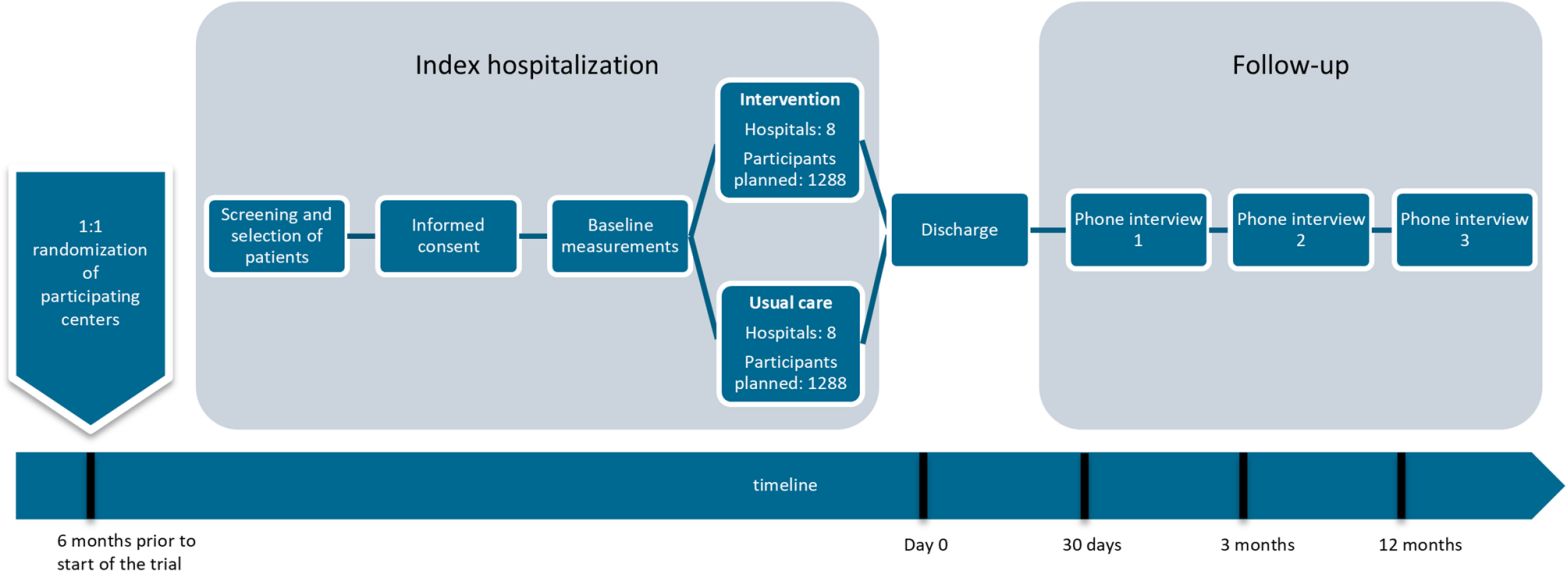
Timeline of the LIMONCELLO-trial

This is an open, parallel-group, cluster randomized controlled trial to evaluate the effectiveness of TMPC compared with usual care. A cluster is defined as a participating hospital. Randomization is conducted six months prior to the start of the trial to allow hospitals enough time for adequate preparation. Randomization is performed 1:1 to either the intervention or usual care arm of the study, stratified by university or general hospital. Sixteen hospitals in the Netherlands participate in the study; seven university hospitals and nine general hospitals. The list of participating hospitals is available online at clinicaltrials.gov (NCT05899114). The total follow-up is one year after discharge, with three telephone interviews at 30 days, 3 months and 12 months after hospital discharge. Figure 1 provides an overview of the study timeline.

### Participants

Consecutive patients are screened for eligibility during hospitalization by the local research team with the support of screening lists generated either digitally or by pharmacy technicians, depending on the hospital’s possibilities. Patients are selected from all hospital wards, except wards where structured medication reviews with transitional multidisciplinary collaboration are already performed by a multidisciplinary team as part of usual care. Table 1 shows the inclusion and exclusion criteria. If a patient’s medical care is primarily coordinated by physicians in secondary care, this patient is excluded, because of the importance of collaboration between primary and secondary healthcare in the intervention. In these cases, the GP in primary care has a limited role during follow-up, which is the case for, e.g., patients receiving intensive oncological treatment. Incapacitated patients can be included in this study if a legal representative provides informed consent.

**Table 1:** Inclusion and exclusion criteria

**Inclusion criteria**
• 70 years or older
• Polypharmacy, i.e., the use of 5 or more regular medications, defined as market authorized medications used for more than 30 days. Topical preparations are excluded from this definition
• Admitted to the hospital through an emergency department (which comprises both the general emergency department and the cardiac emergency department)
• Length of hospitalization of more than 24 h
• Completed medication reconciliation, which is the process of obtaining, verifying and documenting the most complete and accurate list of the patient’s current medication use
• DRA prediction percentage of ≥ 23.0% within 1 year after the index hospitalization [23]
**Exclusion criteria**
• No informed consent by patient or a legal representative
• Participation in an interfering clinical trial
• Elective hospital admission
• Direct admission to the intensive care unit
• An estimated life expectancy of less than three months, which includes patients with palliative treatment at home, direct admission to palliative care or palliative care planned within 24 h after index hospital admission
• Patient or legal representative not able to speak Dutch
• Follow-up of patient primarily by healthcare professionals in secondary care. This refers to situations where the secondary caregiver is in the lead of the medication list of the patient instead of the GP or elderly care physician, for example in the following patient groups:
• patients receiving intensive oncological therapy
• patients in an organ- or stem cell transplantation treatment trajectory
• patients receiving intensive (chronic) psychiatric care, such as patients admitted to a medical psychiatric unit
• patients on renal replacement therapy

Patients at the highest risk of a DRA are identified with an externally validated prediction model called the drug-related readmission (DRA) prediction model [23, 25]. The risk percentage for DRA is calculated on the basis of seven characteristics, each accompanied by a regression coefficient (Table 2). The overall DRA risk percentage can be calculated via the following formula: predicted probability = 1/(1 + e^- (−2.41 + characteristic*regression coefficient)) [23]. The 50% of patients at the highest risk of a DRA are selected for this study, resulting in a cut-off percentage of ≥ 23% risk of DRA within 1 year after the index hospitalization according to the prediction model.

**Table 2:** Drug-related readmission prediction model, characteristics and regression coefficients. Adopted from Snijders et al. 2023 [23]

Characteristic	Regression coefficient
Number of hospitalizations < 1 year prior to index	
*0*	Ref
*1–2*	0.56
≥*3*	0.94
Non-elective admission	0.24
History of hypertension	0.09
History of chronic kidney disease	−0.05
Use of diuretics	0.47
Use of oral corticosteroids	0.23
Polypharmacy	0.57

If a patient is eligible on the basis of the inclusion and exclusion criteria, the patient (or representative) is informed about the study and informed consent is obtained.

### Randomization

The study is cluster randomized 1:1 at the hospital level. Stratification was performed for university or general hospitals because of expected differences between hospital types in standard care. Randomization at the patient level is not preferable, because of potential contamination, as it is expected that there is an (un)conscious learning effect among the responsible physicians and pharmacists by performing the intervention. This might influence the care provided to the usual care group. Cluster randomization is performed six months prior to the start of recruitment by a methodologist via PASS® 2020 Power Analysis and Sample Size Software (version 20.0.9; NCSS LLC. Kaysville, UT, USA).

### Blinding

Owing to cluster randomization at the hospital level and the content of the intervention, blinding is not possible for local research teams (performing baseline data input) or central research teams (performing follow-up data input). Patients are not blinded, since the intervention requires their active participation. However, blinded adjudication of the primary outcome (DRAs) is assured, by assigning an independent team of adjudicators for assessment of the primary outcome.

### Intervention

A pharmacotherapy-team conducts the intervention during the initial hospital stay (i.e. the index hospitalization). This pharmacotherapy-team involves collaboration between physicians and pharmacists, and this multidisciplinary team aims to exchange knowledge. The team is composed of senior staff members with at least one medical specialist and one hospital pharmacist, both of whom are preferably registered as clinical pharmacologists. Other potential team members may include residents in internal medicine or geriatrics, clinical pharmacology trainees or hospital pharmacy residents, who participate with the supervision of a senior staff member. All pharmacotherapy-team members conducting the TMPC are trained in the standardized intervention prior to study initiation during a live training session. This session is recorded to be viewed by new pharmacotherapy-team members as e-learning.

The TMPC intervention is based on the Systematic Tool to Reduce Inappropriate Prescribing (STRIP) methodology [26], which is a structured method for medication reviews developed to improve appropriate prescribing. Prior to the start of the intervention, a medication reconciliation must be performed to ensure that the medication lists are as accurate as possible [27]. Medication reconciliation is a standardized process during hospitalization in the Netherlands, and therefore part of the usual care in all hospitals participating in this trial. The TMPC comprises four elements of which the sequence can be adjusted to meet the needs of the pharmacotherapy-team and patient:

1. A structured pharmacotherapeutic analysis is performed by the pharmacotherapy-team, who identifies pharmacotherapeutic problems such as over- and undertreatment, ineffective treatment, side effects, clinically relevant contraindications and interactions, appropriateness of dosing, and usage problems. Several tools can be used to aid the pharmacotherapeutic analysis, such as the Dutch translation of the STOPP-START criteria [28], and the local clinical decision support system alerts from the hospital information system. Additionally, the tools mentioned in the Dutch national multidisciplinary guideline on 'Polypharmacy in older people' with the module 'Reducing and stopping medication’ (2020) [28, 29], such as the factsheets on deprescribing [30], can be applied during pharmacotherapeutic analysis. The collection of clinical and laboratory data from electronic health records (EHRs) is necessary for optimizing the individual patients’ pharmacotherapy. The GP, elderly care physician (also known as the nursing home physician) and/or community pharmacist are consulted if essential information in the medical or medication record is missing or unclear. Usually, Dutch patients are registered at one GP-practice and one community pharmacy.
2. A transitional multidisciplinary discussion is organized together with the responsible physician in the hospital (medical specialist, medical resident/intern) and one or more members of the pharmacotherapy-team. If a proposed change involves prescriptions from another medical specialist, it is discussed with that specialist. The GP or elderly care physician is consulted to discuss the results of this pharmacotherapeutic analysis and to make arrangements about the follow-up of recommendations after hospital discharge. If community pharmacists should be actively involved in the follow-up/monitoring process of interventions, they will be contacted by the pharmacotherapy-team if deemed necessary.
3. An interview and discussion with the patient and/or legal representative are conducted before discharge by a member of the pharmacotherapy-team. The recommendations described in the pharmacotherapeutic plan are discussed and adjusted, utilizing shared decision-making techniques [31]. Motivational interviewing is used to address problems and needs within the medication plan. To assess the understanding of the patient and/or legal representative the teach-back method is used [32]. If needed, instructions on medication use are explained and adjustments can be made in the pharmacotherapeutic plan by the pharmacotherapy-team after discussion with the patient and/or legal representative.
4. A discharge letter with a detailed pharmacotherapeutic plan is drafted by a member of the pharmacotherapy-team. This letter includes identified DRPs and associated changes made during hospitalization and/or advice on pharmacotherapy (such as initiating, discontinuing, or tapering) after hospital discharge in comparison with the medication used at the moment of admission. Additionally, the rationale for these recommendations and arrangements made for follow-up and monitoring are described. The letter is standardized and has a similar layout in all hospitals, it is addressed to the GP or elderly care physician (it can be found in Supplementary Material 1 ). It can be a separate letter or incorporated into the discharge letter of the admitting specialist. A copy is provided to the patient, community pharmacist and all relevant healthcare professionals involved in the patient’s treatment.

If the intervention cannot be completed during hospitalization due to early discharge of a participant, the remaining elements are carried out after discharge (mostly via telephone). This is only applicable if at least the pharmacotherapeutic analysis has been performed. If a participant is discharged before any element can be conducted, then the intervention will not be carried out after discharge.

### Comparator – usual care

The comparator in this study is usual care, which refers to the entire spectrum of drug-related interventions by various healthcare providers (i.e. physicians, pharmacists, nurses) during hospital admission. This may include a medication review being performed by the prescribing physician or consulting pharmacist in accordance with usual care, which is allowed if it does not involve all the elements of TMPC.

Because usual care may differ across hospitals, the usual pharmacotherapeutic care in all participating hospitals was explored via a detailed questionnaire prior to the start of the study. A translated version of the questionnaire is available upon request.

### Data collection

Baseline data collection is conducted by the local research team during index hospitalization. All local research personnel are trained in data collection through e-learning. At baseline, information is collected about demographics, independence of medication intake, use of medication aids, living situation, falls and fall-related injuries, laboratory results (e.g., electrolytes, renal function; collected in routine care), medical history, reasons for current admission and medication use at both admission and discharge. Furthermore, we collect data through various questionnaires, including EuroQol 5-dimensions 5-level (EQ-5D-5L) [33] for quality of life (QoL), the Katz-6 [34] for activities of daily living (ADL), the Clinical Frailty Scale [35] for frailty, and the iMTA medical consumption questionnaire (iMCQ) [36] for healthcare utilization and costs. In addition, data collection in intervention hospitals includes information about the recommendations given during the pharmacotherapeutic analysis, completeness of conducting TMPC elements and healthcare professionals involved, and duration of TMPC.

Outcome data are collected by a central research team during follow-up telephone calls with participants or their representatives at three time points: 30 days, 3 months and 12 months after discharge from the index hospitalization. Data concerning current living situation, falls and fall-related injuries, independence of medication intake, use of medication aids and current medication use are collected. Furthermore, the EQ-5D-5L, Katz-6 ADL and iMCQ questionnaires are repeated during each telephone interview. All central research employees are trained in performing the follow-up data collection. If a patient or representative cannot be reached after four attempts or if a patient does not wish to answer the questionnaires anymore but still wants to continue study participation, the patient’s GP or elderly care physician is contacted to ask only those questions required to answer the primary research objective.

### Assessment of the primary outcome

The primary outcome is the proportion of patients with a DRA for each study group in the first 30 days after discharge. A readmission is defined as an unplanned hospital admission longer than 24 h that occurs after the index hospitalization. Readmissions are documented on the basis of the information provided during the follow-up telephone interviews with participants combined with information from the patient records in the participating hospital. If a readmission is identified, more detailed information about the admission and medication use is requested from the admitting hospital to determine whether an admission is drug-related via the ‘Assessment Tool for identifying Hospital Admissions Related to Medications’ (AT-HARM10) [37]. This tool is based on ten questions and was validated for use in patients aged 65 years or older [37]. The readmissions are only adjudicated as drug-related if a DRP is the main reason or a significant contributing factor to the admission [37]. The evaluation of drug relatedness in this study is performed by a blinded adjudication committee composed of trained final-year undergraduate or postgraduate pharmacy students in accordance with the validation study. If agreement on the causal association of hospitalization with medication is not reached, a blinded, experienced geriatrician, pharmacist or internist-clinical pharmacologist is consulted as a third assessor to reach consensus.

### Assessment of secondary outcomes

Secondary outcomes are assessed during index hospitalization and three follow-up telephone interviews at 30 days, and 3 and 12 months following discharge from the index hospitalization. The secondary endpoints include the proportion of patients with a DRA at 3 and 12 months after discharge, duration of DRA, time to first DRA, proportion of patients with a drug-related ED visit, proportion of patients with an all-cause readmission and ED visit, healthcare costs, QoL, cost-effectiveness, number of regular medications, ADL, proportion of patients living independently, proportion of patients with falls and mortality. For all binary outcomes listed above, outcomes are presented as both the proportion of patients experiencing at least one event and the total number of events per patient. In the intervention group, the number and type of recommendations in the pharmacotherapeutic plan and the number of implemented recommendations from the pharmacotherapeutic plan are collected as secondary outcomes.

If an admission or ED visit occurs, more detailed information about the ED visit and medication use is collected from the respective hospital to determine whether the ED visit was drug-related via the AT-HARM10 tool [38]. QoL, ADL and healthcare costs are assessed via the EQ-5D-5L, Katz-6 ADL and iMCQ questionnaires, respectively.

The data concerning medication use at admission and discharge is collected from the EHR during index hospitalization. Medication use during the follow-up period is assessed by asking patients or their representatives about the current medication use during follow-up telephone interviews. The number and types of recommendations in the pharmacotherapeutic plan are determined on the basis of the data collected at baseline about the execution of TMPC. The number of implemented pharmacotherapeutic recommendations is assessed by comparing the recommendations with the medication lists collected during follow-up.

If a patient is unable to answer questions concerning their current medication use during follow-up, the community pharmacy is contacted by the central research team to obtain a medication overview. If a patient cannot be reached, their survival status is identified by calling family members, the GP/elderly care physician or a member of the local research team from the participating hospital.

### Data management

Patient data are collected from the EHR and questionnaires; responses are directly entered into a Castor® Electronic Data Capture (EDC) database, version 2024.1.0.3. (Castor, Amsterdam, The Netherlands) [39], hosted by Radboudumc, in which the participant data are automatically pseudonymized using a patient- and hospital-specific number. Data entry is monitored throughout the study period via built-in error notifications in the Castor® database. During the study period, data can only be made available for the study group upon request by a methodologist. The final trial dataset will be available for all data managers involved in the study (from the Radboudumc, Amsterdam UMC and the methodologist of Trialbureau Zorgevaluatie Nederland). After the final data cleaning, the dataset will be made available for analysis to the study group. After the trial is completed, the data will be anonymously published in a repository and made available upon reasonable request. More elaborate information about data management can be found in the study’s data management plan (available upon request).

### Sample size calculation

For the calculation of the sample size, we estimated that the proportion of patients with a DRA in the target group studied is approximately 20% [5]. Previous studies have indicated that multicomponent interventions with a focus on medication optimization can achieve a decrease in DRAs of 6%, i.e., a total readmission rate of 14% [20, 40, 41]. Using a sample size of 1,224 patients in both the intervention and control groups, which are divided into eight clusters of 153 patients in each group, a 6% decrease can be detected from the standard readmission rate of 20% (two-sided, power 80%, alpha 5%). An intracluster correlation coefficient (ICC) of 0.0065 was used, which is based on an estimated ICC between 0.005 and 0.01. With an ICC of 0.01, this group size would be sufficient to demonstrate a one-sided difference of 6% in favour of the intervention. A drop-out rate of 5% is expected [42], requiring a total of 161 patients per cluster to be included, or 2,576 in total. The sample size was calculated via PASS 2020 Power Analysis and Sample Size Software (NCSS, LLC. Kaysville, Utah, USA).

### Statistical analysis

For patient characteristics, descriptive statistics will be used. The inclusion, exclusion and dropout numbers and reasons will be described. The results will be analyzed via intention-to-treat analysis and per-protocol analysis. The statistical framework for this trial is that of a superiority trial. For the primary endpoint, a generalized linear mixed model with a binomial distribution and logit link function will be used for the analysis at the individual level, adjusting for clustering. In the regression model, the treatment group (intervention/control) and the type of hospital (university/general) are used as a fixed effect and the cluster is used as a random effect. A correction with degrees of freedom is applied (between-within method) in connection with the type I error caused by the small number of clusters.

For DRAs over time (30 days, 3 months and 12 months) an unstructured residual covariance matrix will be included, and the time effect and interaction between time and interventions will be evaluated. A similar model will be applied to other binary outcomes. For these outcomes, the proportion of patients with events and the time to first event will be analyzed, similar to the primary outcome. For the analysis of the continuous secondary endpoints, a linear mixed model with the same random effects and correction method will be used. For all linear outcomes, mean differences with corresponding 95% confidence interval (CI) will be presented, and for binary outcomes, odds ratios with 95% CI will be reported. Mortality over time will be evaluated via multi-level survival analysis, and differences between groups will be presented as hazard ratios with 95% CIs. For ordered variables, multilevel mixed-effects ordered logistic models will be used. Statistical analyses will be performed via STATA 18 (StataCorp. 2023. *Stata Statistical Software: Release 18*. College Station, TX: StataCorp LLC), SPSS (IBM Corp. Released 2023. IBM SPSS Statistics for Windows, Version 29.0.2.0 Armonk, NY: IBM Corp) or RStudio (RStudio Team (2024). RStudio: Integrated Development for R. RStudio, PBC, Boston, MA URL http://www.rstudio.com/).

### Economic evaluation

An economic evaluation will be performed to investigate the cost-effectiveness of the intervention compared to usual care. Cost-effectiveness will be expressed in terms of the cost per quality adjusted life year (QALY) gained and via the net monetary benefit approach. Healthcare costs will be assessed via the iMCQ. Lost productivity costs will not be evaluated, as all participants are retired at an age of 70 years or older. Standard cost-prices per item of healthcare consumption will be determined via appendix 1 of the guideline for performing economic evaluations [43]. If standard prices are not available, the full cost prices of items will be used via activity-based costing. The costs of medication use will be derived from the Dutch formulary [44], increased with the cost for pharmacy care and will be based on the medication use confirmed during the follow-up telephone calls or the medication list from the community pharmacy. The costs of the intervention will be calculated through a time measurement of the intervention, which is based on the time spent by the pharmacotherapy-team members on the TMPC elements, and their average salary. For all participants, an overall time estimate will be made and in a selected group an exact time measurement per TMPC element will be executed. QoL will be measured by the EQ-5D-5L, which includes the Visual Analogue Scale. Furthermore, a budget impact analysis will be performed. The short- and midterm affordability of TMPC will be assessed from governmental, health care and insurer perspectives.

### Patient safety and monitoring

Adverse events are not recorded and serious adverse events do not have to be reported to the sponsor and Medical Research Ethics Committee (MREC), as the intervention is a process-optimization of the standard of care. The separate elements used in TMPC during pharmacotherapeutic analysis are all common practices within the Netherlands. Therefore, the risks of the study to the patient are considered negligible. Owing to the low risk of this study, it is not necessary to appoint a Data Safety Monitoring Board/Safety Committee. Information about hospitalizations, ED visits and mortality are collected during this trial, as these are outcome measures of the study. For quality control of the study conduct and data retrieval, monitoring is performed by a qualified monitor through onsite or telephone visits at all participating sites. No interim analysis is executed.

### Patient and public involvement

A patient representative from “Referentenpanel Patiëntenfederatie Nederland” is involved in setting priorities, trial design and conduct of the study, specifically in preparation of the patient information folder, design of the informative video for the LIMONCELLO website and motivational interviewing training. To limit the burden of the study for patients, follow-up is performed through telephone calls, and a priority list for the questionnaires is available. The results of this study will be disseminated to participants through the study website and by involving the patient representative.

### Ethics approval

This study complies with all applicable standards of the International Conference on Harmonization E6 Guideline for Good Clinical Practice [45]. The study protocol and other documentation concerning the study conduct are approved by the MREC Oost-Nederland (protocol number 2022–15907, date of approval: 25–01–2023). Protocol amendments are communicated to relevant parties. A subject insurance cover was not deemed necessary by the MREC.

### Trial status

Trial recruitment started in June 2023 and was anticipated to take 15 months; follow-up was planned to be completed one year after the last inclusion (planned: August 2025). However, recruitment has shown to require more time than initially planned. At the time of manuscript submission (November 2024), data collection was still ongoing. The inclusion period concluded in January 2025, after a total duration of 20 months.

## Discussion

This article describes the design of a multicenter cluster-randomized controlled trial investigating the effect of transitional multidisciplinary pharmacotherapeutic care (TMPC) on the proportion of patients with a drug-related admission (DRA) in hospitalized older people. The aim of this trial is to add evidence on the clinical effectiveness and cost-effectiveness of a multicomponent medication review intervention, called TMPC, compared with usual care in hospitalized older patients who are at the highest risk for DRAs.

Multiple factors contribute to the current lack of clarity about the effectiveness of multicomponent medication review interventions on clinically relevant outcomes. This is partly due to the heterogeneity of hospitals and the target population, making large sample sizes necessary to demonstrate the effects of the intervention [46]. To address this, the LIMONCELLO-study is designed as a large trial. To reach sufficient power, 16 Dutch hospitals distributed across the country participate in this study, representing a substantial proportion of the total 113 hospital locations in the Netherlands [47]. To minimize heterogeneity between the participating centers due to differences in the organization of healthcare services, the study is designed as a single-country trial. However, not all heterogeneity and variability between hospitals is avoidable, which represents a limitation of this study and leads to between-cluster variability. Although the variability is accounted for in the power analysis, this remains a disadvantage of cluster randomization. Nevertheless, we considered cluster randomization to be the most appropriate method of randomization for this trial, because of the decreased likelihood of contamination bias among healthcare personnel compared with individual randomization [15].

To limit the heterogeneity of the trial population, only patients who are at the highest risk of a DRA are included. In previous studies, a relatively general population of older patients with multimorbidity and polypharmacy was selected [42, 48, 49]. In contrast, we select patients at the highest risk of DRAs via the DRA prediction model [23]. To the best of our knowledge, this is the first trial to use the DRA prediction model. We consider this an appropriate model as validation was recently performed [23]. The ideal cut-off value of DRA risk can vary on the basis of the researcher’s objectives, with higher cut-off values leading to higher specificity but lower sensitivity. Initially, the cut-off value of DRA risk for inclusion was set at 27.9% consequently selecting the 30% of patients at highest risk of DRA. However, this threshold was lowered to 23.0% during the recruitment phase of the LIMONCELLO-trial, to increase the prediction model’s sensitivity and thereby enhance recruitment rates. In this way, we were able to balance the feasibility and selection of a high-risk population. We purposively consider patients with cognitive impairment eligible for this study, as they are likely to benefit from the intervention because of frequently occurring medication management problems [50]. Thus, the inclusion of these patients is considered a strength, because they are an understudied population with many remaining knowledge gaps in medication review studies [51]. Explorative subgroup analyses with different patient characteristics will be performed to investigate potential differences between subgroups and assess the generalizability of the study results.

An additional relevant aspect of the LIMONCELLO study design is the multicomponent medication review intervention, which takes the limitations and strengths of previous studies into account. Previous studies have indicated that interventions should include multiple components, such as patient education, improved transition of care and multidisciplinary collaboration [19–21]. Therefore, components focusing on critical moments in the care process are incorporated into the studied intervention [20]. First, medication reconciliation is a requirement to start the intervention. Second, patient education and shared decision-making take place during both the interview and the follow-up discussion with the patient. Third, improved transitional care is ensured because the patient’s GP and community pharmacist are informed through a telephone call and a pharmacotherapeutic discharge letter. In addition, the focus on multidisciplinary collaboration is evident in step three of the intervention: the multidisciplinary discussion of potential pharmacotherapeutic changes in the patient's medication regimen. Although some of the intervention elements are executed in standard clinical practice in the Netherlands, the structured combination of elements is considered innovative.

Successful implementation and proper execution of the described intervention design are necessary to potentially demonstrate effectiveness. Therefore, the pharmacotherapy-team members executing TMPC are trained prior to executing the intervention, including a training in motivational interviewing. Additionally, we integrate pharmacotherapy teams into existing healthcare structures rather than relying solely on research personnel. Through these actions, we aim to improve the level of execution of the intervention and implementation of medication recommendations, which will be evaluated in a process evaluation of the trial.

The strengths of the study, as previously described, also lead to consequent limitations that should be noted. Firstly, selecting a high-risk patient population using the DRA prediction model requires a more detailed search in the EHR to determine study-eligibility, compared to using broader inclusion criteria. This process is more time-consuming for the researchers, and may pose challenges for future implementation. However, previous research indicates that if the goal of a medication review intervention is to reduce drug-related harm, it is essential to select a population at elevated risk of these events [17]. The DRA prediction model was specifically chosen for this purpose. Furthermore, it is designed to be user-friendly and incorporates characteristics that can be relatively easily extracted from the EHR [25]. To address the substantial time investment required for patient selection, automated screening tools incorporating the DRA prediction model are developed and if possible integrated into the EHR during the trial preparation. Secondly, the intervention consists of multiple components, making it more intensive and, consequently, time-consuming. This increases the workload for members of the pharmacotherapy teams, who must integrate these tasks in their current clinical responsibilities. Nonetheless, as previously described, a multi-component intervention is considered essential to ensure potential effectiveness in reducing DRA and other secondary outcomes. Furthermore, to assess the balance between effort and benefit, a process evaluation and cost-effectiveness analysis will be performed. During the trial period hospitals will receive some financial compensation for their time investment. In conclusion, while the design of this study demands additional time investment for patient selection and intervention execution, these efforts are considered necessary to increase the likelihood of achieving meaningful clinical outcomes.

In summary, the LIMONCELLO-trial is a large cluster randomized trial, that aims to add evidence about the effects of TMPC on clinically relevant outcomes in a high-risk subgroup of hospitalized older people. The study design makes the trial outcomes generalizable to the older population in the Netherlands and countries with similar healthcare systems. The outcomes of this study will offer insights into how pharmacotherapeutic care for hospitalized older patients with polypharmacy can be improved.

## Supplementary Information

Supplementary Material 1.

## Data Availability

No datasets were generated or analysed during the current study.

## Abbreviations

ADL: Activities of daily living
AT-HARM10: Assessment Tool for identifying Hospital Admissions Related to Medications
CI: Confidence interval
DRA: Drug-Related reAdmission
DRP: Drug-Related Problem
ED: Emergency department
EDC: Electronic Data Capture
EHR: Electronic Health Record
EQ-5D-5L: 5-level EuroQol-5 domains
GCP: Good Clinical Practice
GP: General Practitioner
ICC: Intracluster correlation
iMCQ: iMTA Medical Consumption Questionnaire
LIMONCELLO: Less Is More: Optimized pharmacotherapy with improved coNtinuity of CarE in hospitaLized oLder people
MREC: Medical research ethics committee
QoL: Quality of Life
STRIP: Systematic Tool to Reduce Inappropriate Prescribing
TMPC: Transitional Multidisciplinary Pharmacotherapeutic Care
