# Supplementary material for: Less Is More: Optimized pharmacotherapy with improved coNtinuity of CarE in hospitaLized oLder peOple (LIMONCELLO): study protocol of a cluster randomized controlled trial
Source: BMC Geriatr. 2025 Dec 24;25:1034. doi: 10.1186/s12877-025-06533-0 (PMC12729224; doi:10.1186/s12877-025-06533-0)
Supplement: Supplementary file 1 — Supplementary Material 1. [file 12877_2025_6533_MOESM1_ESM.docx]

# Format pharmaceutical discharge letter

| Patient name: | Citizen service number: |
| --- | --- |
| Date of birth: | |
| Department of admission: | |
| Reason for admission: | |
| Admission date: | Discharge date: |
| Discharge destination: | |

**Pharmaceutical discharge letter following clinical medication review LIMONCELLO-trial
Concerns:**

Dear colleague,

Your patient is included in a clinical trial called LIMONCELLO (Less Is More: Optimized pharmacotherapy with improved coNtinuity of CarE in hospitaLized oLder peOple).

During hospital admission, a structured medication review was performed, resulting in the (proposed) medication overview below. A careful transfer of information to the general practitioner, community pharmacist and/or elderly care physician (if applicable) is also part of the intervention. Through this letter, we would like to inform you about the conclusions, recommendations and necessary follow-up actions. This overview is a supplement to the medication overview provided at discharge.

**Your patient has been informed about potential medication changes and reasons for this.**

**PATIENT INFORMATION**

Known allergies:

Known side-effects:

Relevant vital signs: *(if applicable: eGFR, sodium, potassium, baseline QTc, relevant TDM, etc.)*

Specific details regarding medication use:

- *Medication management: patient themselves / caregiver / healthcare provider / other, namely …*
- *Swallowing problem: yes / no*
- *Multi-dose drug dispensing system: yes / no*
- *Other medication management problems: yes/no. If yes, which one: …*

**PHARMACOTHERAPY PLAN**

1. **Follow-up advice after discharge**
   *Fill in the medication recommendations (start/stop/change/switch) that have not yet been implemented in the hospital, as well as follow-up checks that need to be carried out in primary care. Note who is responsible for scheduling an appointment for the follow-up checks.*

| **Advice (stop, start, switch, change dose/frequency)** | **Medication** | **Route of administration + dose** | **Indication** | **Comments (justification for the change)** |
| --- | --- | --- | --- | --- |
|  |  |  |  |  |
|  |  |  |  |  |
|  |  |  |  |  |
|  |  |  |  |  |

**Medication advice:**

| **Follow-up – what** | **By whom** | **When** | **Who makes the appointment (patient, GP/specialist elderly care, or community pharmacist)** | **Comments** |
| --- | --- | --- | --- | --- |
|  |  |  |  |  |
|  |  |  |  |  |
|  |  |  |  |  |
|  |  |  |  |  |

**Follow-up recommendations:**

| **Medication** | **Route of administration + dose** | **Comments (justification for the change)** |
| --- | --- | --- |
|  |  |  |
|  |  |  |
|  |  |  |
|  |  |  |

1. **Discontinued medication**

| **New or changed** | **Medication** | **Route of administration + dose** | **Indication** | **Comments (justification for the change)** |
| --- | --- | --- | --- | --- |
|  |  |  |  |  |
|  |  |  |  |  |
|  |  |  |  |  |
|  |  |  |  |  |

1. **Medication list at discharge***Copy from the EHR as close to discharge as possible. Note ‘NEW’ in the first column if a medication has been started during the hospital stay.*

**POINTS OF ATTENTION AND AGREEMENTS ABOUT MEDICATION USE***Note points of attention regarding patient’s medication use that are important to transfer to primary care. Also indicate here if there are any special considerations regarding the medication use or medication management of the patient. If applicable, note any agreements made with the patient regarding their medication use.*

- - Points of attention for primary care: *e.g. involved specialties during admission, reasons to refrain from certain medication advice, consciously accepted medication monitoring alerts for NEW medication, including reason of acceptance.*
  - Agreements made with the patient: *e.g. special instructions for medication use, what to do in case of illness or symptoms, is the patient aware of the follow-up?*

Do not hesitate to contact us if you have any questions.

- For questions about this medication review, we refer you to the team that carried out the medication review: [contact information Pharmacotherapy-team]
- For questions about the trial, we refer you to the LIMONCELLO study investigators: [contact information investigators]
